# Towards a better understanding of the psychosocial determinants associated with adults’ use of smokeless tobacco in the Jazan Region of Saudi Arabia: a qualitative study

**DOI:** 10.1186/s12889-022-13120-0

**Published:** 2022-04-13

**Authors:** Ibtisam Moafa, Rik Crutzen, Bart van den Borne, Mohammed Jafer, Maan Shabi, Ahmed Al-khaldi, Ahmed Abu-Zawah, Hameed Al-jabri, Ismaeel Hedad

**Affiliations:** 1 Department of Preventive Dental Science, Jazan University, Jazan, Saudi Arabia; 2 Department of Health Promotion, Maastricht University/CAPHRI, P.O. Box 616, 6200 MD Maastricht, The Netherlands; 3 College of Dentistry, Jazan University, Jazan, Saudi Arabia; 4 Jazan Dental Center, Ministry of Health, Jazan, Saudi Arabia

**Keywords:** Psychosocial determinants, Smokeless tobacco, Shammah, Saudi Arabia, Qualitative research

## Abstract

**Background:**

Most diagnosed oral cancer cases in Saudi Arabia are in the Jazan region. A common type of smokeless tobacco "Shammah" is prevalent in this region. This study aimed to gain an in-depth understanding of the possible psychosocial determinants of Shammah consumption among adult Shammah users in Jazan region.

**Methods:**

A qualitative study was conducted by means of one-on-one interviews among thirty adult Shammah users. Participants were recruited by means of a purposive sampling technique. Data were collected using a semi-structured interview guide utilizing face-to-face and phone-call interviews. Thematic analysis with hybrid approach was used to analyze the dataset.

**Results:**

Twenty-four sub-codes within four overarching themes were generated. Participants revealed uncertainty related to Shammah composition, how to quit knowledge and Shammah prevention/cessation programs. Shammah use identified as a normal phenomenon in society. Its use was frequently reported in participants’ close network but most users faced family and peers’ disapproval. Some users expressed joy, happiness and focused when using Shammah. Others were disgusted or neutral. Many users believed Shammah causes cancer and tears oral tissues. Others believed it relieves toothache or has no effect. Majority of users were confident to quit and recalled some quitting aids. Toothache, craving, drinking tea and chewing Khat (leaves of Catha edulis plant that causes moderate euphoria) perceived to be triggers to use Shammah. Availability of Shammah, withdrawal symptoms, stress, lack of support, seeing others using Shammah, losing part of routine and toothache were barriers to quit.

**Conclusions:**

Shammah use was associated with uncertainty about Shammah composition and quitting knowledge, social acceptability, influence from family/friends, a range of positive and negative attitudinal beliefs toward its use and high quitting efficacy beliefs. Future interventions targeting Shammah should address the acknowledged triggers and barriers in the present study including the dual use of Shammah and Khat.

**Supplementary Information:**

The online version contains supplementary material available at 10.1186/s12889-022-13120-0.

## Background

Smokeless tobacco (ST) is a global problem affecting more than 300 million people worldwide [1]. ST is a type of tobacco that is not burned or smoked, but that is usually consumed by placing the product against the mucosal sites in the oral or nasal cavities, from which nicotine can be absorbed into the body [2]. In the Eastern Mediterranean region (e.g., Saudi Arabia (KSA), Sudan and Yemen), locally produced ST products (e.g., Shammah, Toombak) are widely used [3]. In KSA, however, data on the prevalence of Shammah use are still scarce.

In the period from 1994 to 2015, the age-standardized incidence rates (ASR) of oral cancer (OC) per 100,000 people in Saudi Arabia ranged from 1.5 in Hail region to 19.6 in Jazan region (highest rate) [4]. A previous retrospective study (1976 to 1995) utilized the available data from the Tumor Registry data at King Faisal Specialist Hospital and Research Centre in Riyadh has identified that 35.4% of the nationally diagnosed cases with OC were from Jazan region [5]. Jazan is a region in the southwest of KSA with a relatively small population of 1,365,110 people compared to the total population of 33,284,101 inhabitants in the KSA (i.e., 4% of the total population of KSA). Previous reviews have linked the high incidence of OC in this region with Shammah dipping [6, 7]. Shammah is a traditional type of ST made of mixture of tobacco, pepper, oils, lime, ash, coloring and flavoring materials [6]. Shammah is placed (dipped) in the buccal vestibule (space between the internal cheeks’ tissue and teeth), labial vestibule (between the internal lip’s tissue and teeth), below and above the tongue. After few minutes of Shammah dipping, the user spits it out. Shammah.

In 2005, WHO has published a report of global data on the incidence of OC where the female population of KSA reported a higher ASR of OC (3.3 to 6.8 per 100,000 people) compared to the male population (≤ 3.2 per 100,000 people) [8]. The reported findings were further illustrated in a study that collected data from patients visiting King Fahd Hospital and Prince Mohammed Bin Naser Hospital in Jazan region in the period from 2012 to 2016 [9]. The study found that 57.5% of all OC cases were females and 42.5% were males. Which contrasts the findings reported from a previous case–control study with data limited to the OC cases diagnosed in 2014 at King Fahd Hospital in Jazan region. The diagnosed OC cases in the latter study were slightly higher among males; 18.74% in males and 14.58% in females [10].

Despite the high prevalence of Shammah usage in Jazan, the attention paid towards curbing Shammah use in terms of research, interventions, policy formulation and implementation are far from optimal [9]. Moreover, no study has been conducted before to explore possible reasons of using Shammah in the Jazan region, including women.

In order to understand Shammah usage behavior, the related psychosocial factors must be identified. The focus on these determinants is related to their ability to be changed and because of their influence on behavior. Multiple psychosocial theories have explained the possible determinants of comparable health-risk behaviors (e.g., smoking). Based on the Reasoned Action Approach (RAA), individual’s attitude, subjective norm, perceived and actual behavioral control predict the intention to change behavior [11]. Also, constructs from the Social Cognitive Theory (SCT), outcome expectancies and self-efficacy are significant determinants of behavior change [12]. Outcome expectations refer to the belief that Shammah benefits or harms people’s health while self-efficacy refers to the people’s confidence in their ability to quit Shammah usage. The Integrated Change model (I-change model) considers the degree to which an individual knows about his behavior, the knowledge about the effect of using Shammah and its health threat, the beliefs about potential vulnerability to OC and the internal and external prompts as factors influencing an individual’s pre-motivational phase of behavior [13]. These determinants refer to psychosocial factors in general and need to be explored in-depth to understand their relevance to understanding a specific behavior (e.g., Shammah use). The integration of these theories uncovers the aspects that are under-represented in each of these separate theories. Moreover, it brings a rich understanding of the possible psychosocial determinants related to using Shammah from different perspectives. The operationalization of the behavioral determinant attitude is different among these theories. For instance, in the RAA, attitude entails two dimensions: instrumental (cognitive) and experiential (affective). While in the SCT, the emotional aspect of attitude is not covered, and instead, attitude is operated by the outcome expectation that is a subjective estimate of the consequences of doing a particular behavior. Another example is self-efficacy; we draw on the evaluation guidance provided by both the SCT and the RAA to enable a thorough and complementary assessment specification of the individual belief about his ability to perform the healthy behavior.

Therefore, the objective of the present study was to gain in-depth information about the possible psychosocial determinants of Shammah usage behavior among adult Saudi current Shammah users. Knowledge and understanding of these determinants are expected to provide targeted information for the development of effective interventions for Shammah use prevention and cessation.

## Methods

### Study design and subjects

In the report of this cross-sectional qualitative study, we adhered to the consolidated criteria for reporting qualitative research data (COREQ) [14]. The participants were selected using a purposive sampling technique to guarantee information-rich and relevant data related to the research problem. The sample was recruited from the network of the authors and participants’ network and was interviewed by the two of the authors face-to-face and via telephone as they were outside the region. We followed hybrid forms of saturation. We considered that we reached data saturation if we achieved both: a priori thematic saturation and inductive thematic saturation. The prior thematic saturation achieved when the predefined codes are sufficiently represented in the data. While the inductive thematic saturation is achieved when there is no new codes/themes anymore. The interviews scheduled at a suitable time and location for participants to avoid any distraction such as at Dental clinics of Jazan University or at their own home. The choice of home environment underpinned by the familiarity with the environment that would help participants to relax and lead to a more productive interview [15]. Recruitment eligibility criteria included Saudi in the age range of 18–80 years, living in Jazan region, and using Shammah. Participants were briefed about the nature of the study and how their contribution would help in designing an effective intervention to tackle the OC associated risk factor Shammah use in the region. They were informed about their right to refuse audio-recording or to withdraw at any point in time. Also, participants were also assured that all their information would be treated confidentially, and was pass secured, and that only authorized personnel would have access to the data. Further, participants were informed that any information they provided and that would be used in reports or publications would be anonymized. Every participant signed the informed consent before starting the interview. For participants who were interviewed in a phone call, signed consent forms were received from participants email/ or from their adult daughters/sons in case of illiterate participants.

### Data collection instruments and measures

Data were collected using semi-structured interview guide. Initially in 2018, we only interviewed the female users of Shammah due to limited feasibility and resources at that time. In 2020, the authors expanded the study to include male users of Shammah. Including male participants has enriched the study findings, deepened our understanding of the determinants of Shammah use and brought new insights that were not previously reported by female participants. The interview guide consisted of ten key open questions with following probes to guide participants talk as shown in the following examples:

1. What do you think about Shammah?

*Probe:*

- What else comes to mind when you think about using Shammah?

The full version of the interview guide can be seen in the supplementary file 1 (See Supplementary File 1). The semi-structured interview flexibility allowed for the discovery of new information that was important to participants, which may not have previously been though of by the interviewer. The interview guide was in the local community dialect of Arabic language and was pretested among five participants from the target group to assess if the questions were clear and understandable. Participants addressed the clarity of the interview questions. Most of the interviews were audio-recorded, but eight participants refused the recording. One reason was due to the cultural sensitivity with respect to recording female voices. The authors did data transcription simultaneously during conducting the interviews for those participants who refused audio recording. The authors verbatim transcribed interviews.

### Development of a coding tree

First, a coding tree was generated for each gender to allow the emergence of novel codes based on the independent perceptions of each gender. Saudi Arabia has a unique socio-cultural context in which a cross-gender communication is highly subjected to sociocultural constraint. For instance, cross-gender interviews were not feasible when the study was conducted. Participants preferred to be interviewed by the same gender and each gender carried some unique perceptions toward Shammah use. The coding tree was based on the four theoretical constructs (knowledge, attitude, subjective norms, and confidence on ability to quit skills). Two debriefiers (independent researchers) with methodological experience and training on qualitative research provided external check and reflection on the data analysis to assess if the coding process was maintained and whether the data accurately captured the participants’ perspectives. The peer debriefing was done irrespective of the gender. We utilized peer debriefing to enhance the validity of the study findings (triangulation). The generated coding tree was returned to the participants who were available and agreed to recheck the themes. We asked them to check the extent to which the generated themes were accurate and confirmative to their words. Second, the male coding tree (based on the 15^th^ interviews) and the female coding tree (based on the 14^th^ interviews) were merged into one communal coding tree by the research team using established consensus method [16]. The 16^th^ male interview was coded using the communal coding tree.

### Data analysis

For each gender, data were analyzed using thematic analysis with an adapted version of the hybrid (deduction-induction) approach [17]. This methodological approach integrates theory-driven codes based on the tenets of psychosocial theories with data-driven ones (emergent codes). Stage 1: developing the codes template (pre-set codes) based on the research question and the theoretical constructs (knowledge, attitude, subjective norm, self-efficacy, skills). Stage 2: familiarizing with the dataset. Stage 3: analysis of the data applying the template of codes to the data set. All the responses were word-level coded in a systematic way related to the pre-defined codes template. Stage 4: identifying the emergent codes from analyzing the data. When new issues came forward, they were linked to the pre-defined themes. Data which fell outside the potential alternatives (pre-set codes), was designated to a new theme. Data were coded manually and analyzed on the Arabic transcripts to avoid potential distortion or loss of data.

## Results

### Participants’ characteristics

Thirty interviews were conducted of which only two interviewees did not complete the interviews because of their busy schedule but they agreed that their partial interview data were included in the study. Table 1 shows the demographic characteristics of the participants in the study. Participants ranged in age from 18 to 80. The majority of participants were educated at varying degree of educational levels. Six participants were not educated and many participants were not employed. They were housewives, students or looking for jobs. Participants were from seven governorates in Jazan region, ‘Sabia’; ‘Al-Aridah’; ‘Abu-Arish’; ‘Al-Ahad’; ‘Baish’; ‘Damad,' and ‘Gezan’. Most participants were using Shammah only and 9 male participants were using it with Khat (Chewing leaves of Catha edulis plant that causes moderate euphoria and excitement prevalent in Ethiopia, Somalia and the Arabian Peninsula). Most participants reported they were using Shammah for more than three years.

**Table 1:** Demographic characteristics of 30 participants

**Characteristic**	**Frequency** **(M**^a^** = 16; F**^b^** = 14)**
**Age**	
(18–36)	18
(37–48)	5
(49–60)	6
(61 +)	1
**Education**	
Illiterate	6
Elementary	6
Intermediate	3
Secondary	7
University/Higher degree	8
**Marital status**	
Single	10
Married	20
**Employment**	
Housewife	12
Student	5
Full time job	7
Looking for job	6
**Governorate**	
Sabia	9
Al-Aridah	6
Abu-Arish	5
Baish	4
Damad	2
Gezan	2
Al-Ahad	2
**Initiation of use**	
Less than three years	3
Equal or more than three years	27
**Exposure time**	
Every hour	14
Once per day	11
Weekly	5
**Site of use**	
Everywhere	14
Below/ above tongue	12
Unilateral: Right/left side	4
**Using Khat**	
Use Shammah with Khat	9
Use Shammah alone	21

^a^male, ^b^females

## Knowledge about Shammah

### Uncertainty about Shammah composition

Participants revealed uncertainty about Shammah’s composition. Few participants acknowledged that Shammah is made of tobacco mixed with salts and other substances.

*"It's made of dust this is what I know", “I really don’t know. I just found it at groceries”, “Just Shammah. I don’t know”, “It is a substance produced from tobacco mixed with salt and other products” –* Interviews 1, 4, 9 and 5

Whereas nine participants acknowledged that Shammah made of grinded trees and dust mixed with substances as salt, glass, Dogdogah, Hashar, and Sorrat. The last three ingredients as described by participants were trees, but they did not know what exactly they are or how these trees look like.

*"I don't know what type of trees but they say its name is (Dogdog) but I don't know what it is. They grind it and sell it to people”, “They say it’s (Hashar). They bring it and grind it”, “and the other types I heard that they add salt to it and grind it in the grinders”-* Interviews 4, 6 and 7

### Types of Shammah

Majority of participants had mentioned that there are different types of Shammah: black; white; yellow; green and red and that they differ from each other in color, ingredients, taste and the prospected user. Some participants favored yellow Shammah over the rest of types, and the reason was owing to its smooth consistency.

*“There are different types. White and red one”, “it’s the same as other types of Shammah. It only differs in color”, “The yellow one is good”, “There is black one it’s for the foreigners like Sudanese”-* Interviews 4, 6, 8 and 10

### Uncertainty about Shammah prevention and cessation programs

All participants except one were not aware about the prevention/cessation programs for Shammah available in the region.

*“I didn’t hear about them”, “What are they and where they are. I don’t know anything about them”, “Guide me, who should I seek to assist me in quitting?”* - Interviews 3, 8 and 14

### Uncertainty about know how to quit

Majority of participants acknowledged uncertainty about how to quit Shammah.

*“I don’t know how to quit”-* Interview 3.

## Attitude toward Shammah

### Feelings

Participants expressed many feelings toward Shammah. These feelings included the feeling of happiness, joy, focused, craving, disgusting as well as neutral.

*“You feel happy and joy but if you don’t use it, you’ll feel sad and boring”, “It’s dirty. It’s not good”, “Even myself, I don’t like it.”, “I don’t feel anything. Its normal like other people”-Interview 2, 3, 6 and 7*

### Cognition

Participants had a combination of the following attitudinal beliefs toward Shammah: causes cancer; relieves toothache (remedy/cure); tears oral tissues and has no effect on oral/systemic health.

*“It is a remedy that I used to do it and now I cannot stop”, “It causes cancer”, “I have never heard anything about it. It causes nothing because you don’t swallow it”, “…tears your mouth” -* Interviews 8, 9, 10 and 11

## Subjective norms

### Social acceptance

The use of Shammah seems to be indigenous in Jazan region. Participants frequently reported social acceptance of Shammah use in Jazan and that it symbolizes a social bond between the individuals.

*“It’s normal to find someone using Shammah in every house in Jazan…most people* *in Jazan region are using it", “It is considered as a social link between the colleagues and friends."* - Interviews 4 and 16

### Shammah consumption by family and friends

Participants reported that more than one member in their family (‘Parents, grandparents, daughters, sisters, cousins’ and ‘friends’) were using Shammah.

*“My dad and two of my daughters and sisters using it”, “Elder people and relatives are using it*” - Interviews 4 and 6

### Greater disapproval of Shammah consumption by family and friends

Only four participants reported having family member supporting Shammah use. Despite Shammah’s commonness, participants reported many important members in their family and friends were disapproving their usage of Shammah.

*"Nobody agrees to use it. Nowadays, even children they tell you to stop using it”, "My sons disapprove”, “My husband tells me to quit it but I tell him to quit smoking too because he is addicted to smoking”-*Interviews 2, 6 and 12

## Confidence and skills to quit

### Enabling factors

Sixteen participants were confident in their ability to quit whenever they want. Participants recalled some aids that enabled them to quit in the past for a short time, and they believed it can help them to quit in the future. These aids can be classified into three categories:

#### Using ‘natural and behavioral alternatives

Fasting Ramadan (9^th^ month of Islamic calendar observed by all Muslims as month of fasting and abstinence from food, drinks and smoking/smokeless tobacco use from sunrise until sunset). Also, changing friend group, changing lifestyle, having support from friends and family, use work as distraction, ginger, cloves, coffee, gum, Miswak (teeth cleaning twig made of Salvadora Persica which is advocated by Islamic hygienical jurisprudence) and cardamom (strong aromatic spice made of genera Elettaria plants commonly used in food and drink).

“I can quit it whenever I want, I use ginger when I don’t have Shammah”, “My mother was chewing gum to distract herself”, “When I am fasting, I don’t use it”–Interview 7, 3, 8

#### Tackling the trigger of using Shammah

Shammah usage was addressed by some participants as a hard to quit habit. The longer period of using Shammah and the high exposure to Shammah in terms of site and time, e.g., every hour and everywhere in the mouth, were common features among participants who described Shammah usage as a habit. Participants identified craving; toothache as well as drinking tea as triggers to use Shammah. Additional trigger to use Shammah that was recited only by the male participants was using Khat.

“The craving”, “When I have toothache, I use it”, “With drinking tea. Shammah adds special taste”, “I use it mainly while chewing Khat” – Interviews 7, 8, 17 and 22

For some participants, treating toothache perceived to be enabler for their quitting.

*“… when my tooth treated”*- Interview 10

#### Using medicine

Some participants mentioned they heard about tablets and patches that can help in quitting.

*“They said there are tablets and patches that help to fight Shammah and quit it”,* - Interviews 5

### Impeding factors

Participants addressed withdrawal symptoms (dizziness, headache, nervousness, laziness); toothache; stress; Shammah availability; seeing others using Shammah; lack of support and feeling of losing part of daily routine as the factors that make Shammah quitting difficult.

*“It’s available in markets and everywhere which makes quitting difficult”, “But when I saw other females using Shammah, I craved for it and returned to use it”, “I become dizzy and less conscious about what surrounds me”* – Interviews 2, 12, 11

## Discussion

The study findings revealed that the use of Shammah among the adults was associated with a limited knowledge of Shammah, negative and positive attitudinal beliefs towards Shammah, having family/friend uses Shammah, lack of actual control skills, lack of social support, using Khat, perceived social acceptance of Shammah use and lack of awareness about tobacco prevention/cessation programs. Most Shammah users specifically females, had a low understanding of the Shammah composition and its consequences on their health. The reported low knowledge of Shammah might be related to participants’ lower education. Almost half of participants especially the females were either illiterate or having only elementary school education. The relation between the knowledge of ST and the educational level supports previous studies [18, 19]. A study conducted among university students found that non-users significantly scored higher in knowledge in comparison to ST users [20].

Many female participants shared a positive view toward using Shammah as a remedy. The use of Shammah as a remedy to cure a tooth pain was also evident in Southeast Asian countries [18, 21]. Many participants shared unfavorable beliefs toward using Shammah, but these beliefs seem not sufficient for them to not use it. This is in line with a previous study that comprehensively explored factors associated with ST usage and cessation [22]. Many participants in the study linked their initiation of Shammah use to their older relatives’ inducement. The influence of a family member (subjective norm) who uses ST on the initiation of another family member to use ST was shown in previous studies [23, 24]. However, two meta-synthesis found that subjective norm was the second in predicting the intention after behavioral control [25, 26]. This is clear from this study as most participants who had the intention to quit did not have the actual Shammah quitting skills despite the disapproving pressure exerted by their families and friends.

Lack of social support from family and friends perceived by most male participants as an impeding factor seems to make quitting difficult. Evidence showed that social support helps individual in dealing with stress, quitting tobacco successfully and in preventing relapse [27]. Another impeding factor that represented a barrier against quitting Shammah among participants was the availability and accessibility to Shammah. Evidence showed a strong association between the accessibility to ST points of sale, the use of ST, the exposure to positive tobacco norms and the difficulties in quitting [28]. Using Khat was reported among male participants who considered it a prompt to Shammah use. The dual use of Khat and tobacco was reported in a systematic review that investigated the use of tobacco among Khat users [29]. In line with our study findings, previous evidence reported that Khat chewing can act as a gateway to the initiation of tobacco use [30, 31]. This may reflect on probable interaction between Khat use and tobacco use that challenges tobacco cessation and prevention efforts [29].

The perception of treating toothache as enabling factor to quit Shammah as recalled by some participants was also evident in previous studies and may give us a hint about possible access disparities to oral health services in the region [18, 21]. Fasting Ramadan was recited by many participants as enabler for Shammah quitting. The month Ramadan, especially in Islamic countries, affords unique environment clear from most of public influences that trigger tobacco use, e.g., no individual smokes during the daytime throughout the month. Therefore, tobacco cessation interventions can benefit from this month in encouraging tobacco users to start cessation efforts duing Ramadan. Several studies reported that smokers who were visitng tobacco cessation clinics have succesfully quitted smoking during Ramadan [32, 33].

Participants’ lack of knowledge about available ST prevention and cessation programs is a cause of concern. Further, there is no published evidence about the interventions used for Shammah cessation in the Jazan region. Which may question the effectiveness of available Shammah prevention/cessation programs in the region. Hence, there is a need to inspect the current programs and assess the opportunity to develop evidence-based programs targeting Shammah use considering effective dissemination strategies. ST cessation services such as behavioral counseling and easily accessible methods as Quitline counseling and web-based service, with or without pharmacotherapy proven to be successful in solving the accessibility to quitting centers [34]. Which might be utilized for the participants who had accessibility issues to healthcare services. Combining the oral screening with Quitline support can have more benefit in quitting ST than using each way separately [35]. Organized mass-reach community health campaigns with multiple-media forms are potential approach with high reach capacities. This approach can be fruitful in raising public awareness, health literacy and shaping the social norms toward Shammah use [36].

To the best of our knowledge, this is the first study that explored the determinants of using the smokeless tobacco “Shammah” from the perspective of current Shammah users of both genders. Participants were recruited from seven governorates in Jazan and therefore Shammah users in the other governorates may have different perceptions. Thus, the interpretation of the study finding should consider its restriction to the included seven areas in Jazan region. However, the hallmark of the qualitative study is to provide in-depth understanding and explanations rather than generalizing the findings. One of the challenges confronted during data collection was participants’ acceptance to record the interview. Some participants strongly opposed audio recording. The interviewers had to conduct and transcribe the interviews simultaneously. Another challenge was about the setting of the interview. Although participants have given the choices whether to meet at Jazan dental clinic or their home, some participants favored to do the interviews through phone calls, agreed to record the call. In the phone interview, the interviewer is not able to see the participants’ body language. This might result in loss of visual and contextual clues, which is mainly of great importance in the anthropologic method of qualitative study design, e.g., participant-observation [37]. However, there is limited evidence that the quality of the interpretation of data is compromised when information is collected by Phone [38].

## Conclusions

Shammah use was associated with uncertainty about Shammah composition and quitting knowledge, social acceptability, influence from family/friends, a range of positive and negative attitudinal beliefs toward its use and high quitting efficacy beliefs. Future interventions targeting Shammah should address the acknowledged triggers and barriers in the present study including the dual use of Shammah and Khat.

## Implications for Practice

Dentists and health professionals are in positions requiring them to have contact with the public every day. They should discuss concerns regarding (the consequences of) Shammah use and provide guidance and support for the Shammah users in quitting. Mass-reach health campaigns can be organized to educate public about the implications of Shammah addiction, to refer them to supporting sites/services and to denormalize Shammah use as a strategy to improve the public health. Authorities need to consider more enforcement of strict policies against Shammah availability. Fasting Ramadan enabled many study participants to temporarily quit Shammah and thus, health educators can take advantage from this month to encourage quitting and sustain individuals in abstinence from all tobacco products. It would be interesting to expand the study over the uncovered governorates to explore their beliefs toward Shammah and to see whether it is similar or differs from what was found in this study. The findings revealed an ambiguity around effectiveness of available active Shammah prevention/cessation programs in the region. Hence, there is a need for evidence-based programs targeting Shammah use.

## Themes

For the female coding tree, which was based on 14 interviews, data saturation was reached by the 12^th^ interview. For the male coding tree, which was based on 15 interviews, data saturation was reached by the 14^th^ interview. Finally, the communal coding tree was confirmed by the 16^th^ male interview. The male and female coding trees were comparable. The interviews’ outcomes are described following five predefined codes referring to psychosocial determinants: *‘1. knowledge about Shammah’; ‘2. attitude toward Shammah’; ‘3*. *subjective norms toward Shammah’* as well as ‘*4*. *confidence on one’s ability and skills to quit Shammah*’. Each code had several sub-codes (twenty-four). The coding tree in supplementary Fig. 1 provides an overall presentation of the predefined and generated codes and sub-codes (See supplementary Fig. 1).

## Supplementary Information

**Additional file 1:** **Supplementary File 1.** Semi-structured interview protocol.

**Additional file 2:** **Supplementary Figure 1.** Coding Tree.

## Data Availability

Data of the study are available on reasonable request from the corresponding author. Due to the sensitivity and privacy of participant’s identity, data are not available publicly.

## Abbreviations

ST: Smokeless Tobacco
KSA: Kingdom of Saudi Arabia
ASR: Age-Standardized Rate
OC: Oral Cancer
RAA: Reasoned Action Approach
SCT: Socio-cognitive Theory
