# Supplementary material for: Towards a better understanding of the psychosocial determinants associated with adults’ use of smokeless tobacco in the Jazan Region of Saudi Arabia: a qualitative study
Source: BMC Public Health. 2022 Apr 13;22:732. doi: 10.1186/s12889-022-13120-0 (PMC9006419; doi:10.1186/s12889-022-13120-0)
Supplement: Supplementary file 1 — Additional file 1: Supplementary File 1. Semi-structured interview protocol. [file 12889_2022_13120_MOESM1_ESM.docx]

**The Semi-structured Interview Guide**

1. What do you know about Shammah?
2. What do you know about Shammah Prevention/Cessation intervention programs in Jazan?
3. What do you think about Shammah?
4. How do you feel toward using Shammah?
5. How do your family, friends, social network perceive using Shammah?
6. How confident are you in your ability to quit Shammah?
   1. In your opinion, what factors/ circumstances enable you to quit Shammah?
   2. In your opinion, what factors/circumstances make it hard/impossible for you to quit Shammah?
7. Can you describe your experience with Shammah?

*Probes:*

1. When did you start using it?
2. How many times per day? Which site in your mouth? For how long?
3. Which circumstances trigger you to use it?
4. Do you have anything to add or to suggest?
5. Any recommendation about who should I visit to learn more about my questions?
