# Supplementary material for: Towards a better understanding of the psychosocial determinants associated with adults’ use of smokeless tobacco in the Jazan Region of Saudi Arabia: a qualitative study
Source: BMC Public Health. 2022 Apr 13;22:732. doi: 10.1186/s12889-022-13120-0 (PMC9006419; doi:10.1186/s12889-022-13120-0)

Figure 1. Communal Coding Tree for the Psychosocial Determinents of Adults' Shammah Usage in Jazan Region, Saudi Arabia

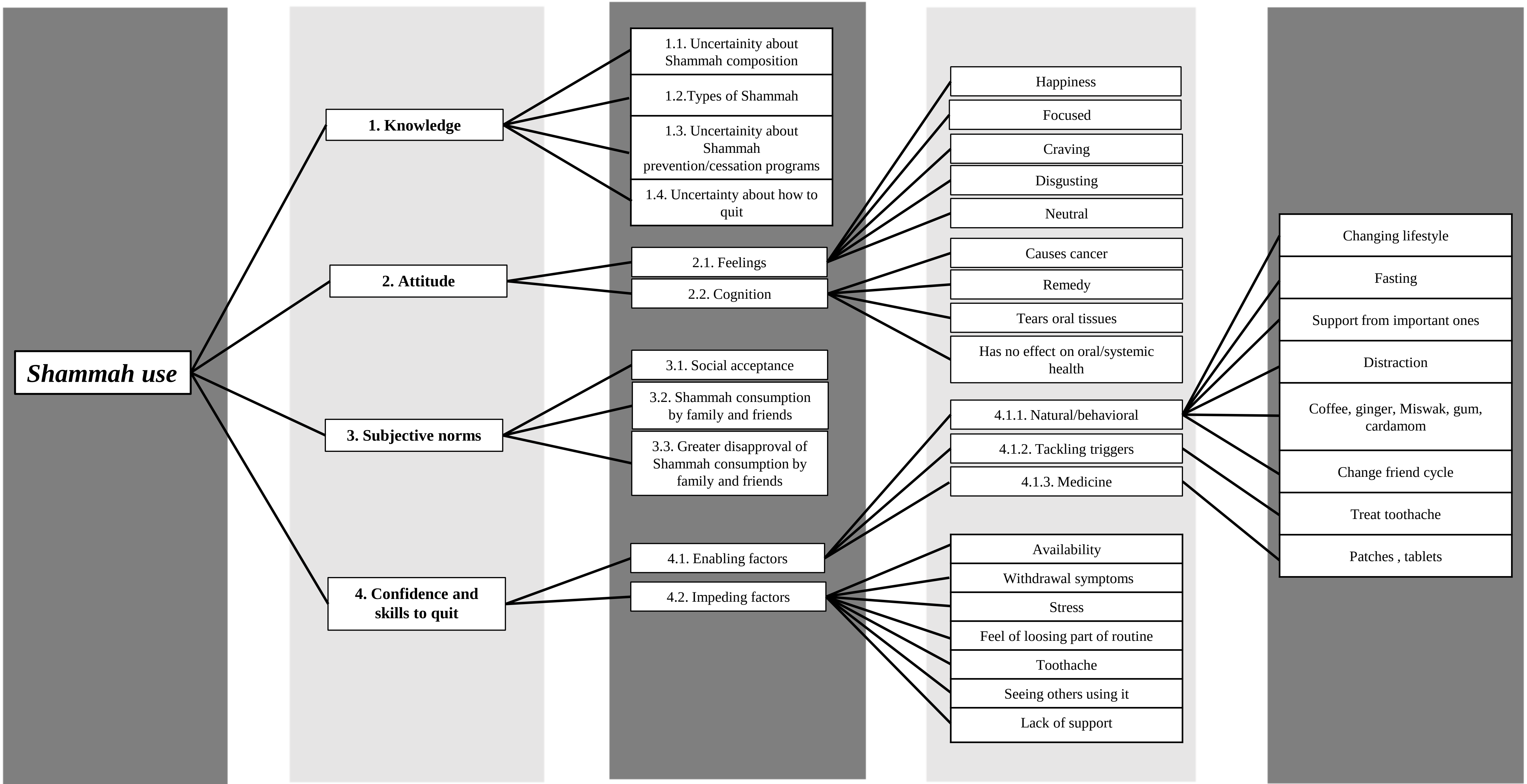

Supplement: Supplementary file 2 — Additional file 2: Supplementary Figure 1. Coding Tree. [file 12889_2022_13120_MOESM2_ESM.pdf]
